# Exposure and impact of a mass media campaign targeting sexual health amongst Scottish men who have sex with men: an outcome evaluation

**DOI:** 10.1186/1471-2458-13-737

**Published:** 2013-08-08

**Authors:** Paul Flowers, Lisa M McDaid, Christina Knussen

**Affiliations:** 1 Department of Psychology and Allied Health Sciences, School of Health and Life Sciences, Glasgow Caledonian University, Glasgow G4 0BA, UK; 2 MRC/CSO Social and Public Health Sciences Unit, 4 Lilybank Gardens, Glasgow G12 8RZ, UK

**Keywords:** Gay men, Mass media, Sexual health, HIV test, Exposure

## Abstract

**Background:**

This paper explores the exposure and impact of a Scottish mass media campaign: Make Your Position Clear. It ran from October 2009 to July 2010, targeted gay men and other men who have sex with men (MSM), and had two key aims: to promote regular sexual health and HIV testing every 6 months, and to promote the use of appropriate condoms and water-based lubricant with each episode of anal intercourse.

**Methods:**

A cross-sectional survey (anonymous and self-report) was conducted 10 months after the campaign was launched (July 2010). Men were recruited from commercial venues. Outcome measures included use of lubricant, testing for sexually transmitted infections and HIV, and intentions to seek HIV testing within the following six months. Linear-by-linear chi-square analysis and binary logistic regressions were conducted to explore the associations between the outcome measures and campaign exposure.

**Results:**

The total sample was 822 men (62.6% response rate). Men self-identifying as HIV positive were excluded from the analysis (n = 38). Binary logistic analysis indicated that those with mid or high campaign exposure were more likely to have been tested for HIV in the previous six months when adjusted for age, area of residence and use of the “gay scene” (AOR = 1.96, 95% CI = 1.26 to 3.06, p = .003), but were not more likely to be tested for STIs (AOR = 1.37, 95% CI = 0.88 to 2.16, p = .167). When adjusted for previous HIV testing, those with mid or high campaign exposure were not more likely to indicate intention to be tested for HIV in the following six months (AOR = 1.30, 95% CI = 0.73 to 2.32, p = .367). Those with no campaign exposure were less likely than those with low exposure to have used appropriate lubricant with anal sex partners in the previous year (AOR = 0.42, 95% CI = 0.23 to 0.77, p = .005).

**Conclusions:**

The campaign had demonstrable reach. The analysis showed partial support for the role of mass media campaigns in improving sexual health outcomes. This suggests that a role for mass media campaigns remains within combination HIV prevention.

## Background

Gay men and other men who have sex with men (MSM) suffer particular sexual health inequalities and are the principal group at greatest risk of acquiring HIV in the UK [1]. They are also disproportionately affected by a range of sexually transmitted infections (STIs), particularly syphilis and gonorrhoea [2]. Responding to the sexual health needs of MSM represents a key public health priority and there is increasing global demand for evidence-based policy and practice with regard to the best approach to sexual health promotion for this group. Although there has been a trend to frame, and inevitably fund, HIV prevention in biomedical terms [3], there remains a clear role for behavioural scientists and health psychologists in informing intervention design and development, in contributing to understanding the process of behaviour change, and, as reported here, in evaluating interventions focussed upon sexual health promotion.

There is global consensus regarding the need for ‘combination’ HIV prevention [4-6]. This is usually conceptualised as a combination of different types of intervention (typically categorised as biomedical, psychological/behavioural and structural) straddling a spectrum of different levels of delivery (e.g., somatic, individual, dyad, group, community, societal, cultural). The evidence for the success of such interventions is governed not only by effectiveness, but also by existing standards of research methods and designs [7]. Thus compared to biomedical interventions, fewer trials concerning either psychological/behavioural or structural interventions get funded and their potential to contribute to the evidence base is systematically negated [3]. In relation to the high quality evidence that does exist, questions remain about key choices in the measurement of effectiveness.

One longstanding and arguably central tenet of HIV prevention, working at the psychological/behavioural and structural levels and delivered to the community, has been the mass media campaign or, more recently with nuanced targeting, the social marketing approach [8,9]. There is some evidence across a range of populations and settings that supports the effectiveness of these approaches with respect to HIV prevention; however, effect sizes tend to be small to moderate, and short lived [10,11]. A dose–response effect to mass media messages has also been demonstrated in various settings and with various international populations, with increased exposure to mass media resulting in increased positive behavioural change [12]. These findings are echoed across the wider literature regarding mass media interventions targeting health behaviours within a range of other health conditions [13].

Mass media approaches represent particularly complex interventions and are notoriously difficult to evaluate [7,14-16]. One central re-occurring problem within this field is the lack of studies featuring randomised control trials (RCTs) or other experimental designs involving control groups. In part, these limitations relate to the demand characteristics of mass media campaigns themselves: by design they aim to maximise spread and saturation within a population. Further, evaluations of campaigns are often subject to compromise from the outset, given problems with funding and external pressures to implement or roll out campaigns within a few months, leaving researchers insufficient time to obtain baseline pre-exposure measures. Noar et al. [11] highlighted that only 30% of the published mass media campaign evaluations targeting HIV used robust evaluative designs, and most used either within-group pre-test-post-test designs (38%) or post-test only designs (32%). The key problem with these designs is that it is difficult to disentangle campaign effects from other explanations such as, for example, reverse causality, differences within sample characteristics, secular trends and/or historical events [17]. A further systematic review of social marketing interventions to increase HIV/STI testing uptake (specifically amongst MSM and male-to-female transgender women) showed that multimedia social marketing campaigns had a significant impact on HIV testing uptake: odds ratio (OR) = 1.58, 95% Confidence Intervals (CI) = 1.40 to 1.77 [18]. This supports the results of a previous systematic review addressing mass media and HIV testing in various populations [19]. However, the campaigns identified were not found to be effective in increasing STI testing uptake (OR = 0.94, 95% CI = 0.68 to 1.28) and, in only including RCTs and other controlled research designs, it identified only three studies [20-22], none of which explicitly utilised theoretical frameworks.

Given the strict inclusion criteria of the systematic reviews outlined above, it is worth examining the findings in relation to evaluations of mass media interventions that are similar to that reported here. Martínez-Donate et al. [23], for example, report on a social marketing campaign, ‘Hombres Sanos’, which targeted behaviourally bisexual Latino men in the USA. It used a variety of print materials in a variety of locations with radio ads and local activities in clubs. Behaviourally bisexual men were recruited through multiple cross-sectional surveys. Of these, 87% reported exposure to the campaign. Details of self-reported behavioural changes as a result of the campaign were assessed. Behavioural changes resulting from exposure to the campaign included around 20% claiming it had made them use condoms. A further 10.8% reported that as a result of the campaign they had sought an HIV test, while 9.2% reported that they had sought an STI test. Similarly, Plant et al. [24], in an evaluation of a social marketing campaign addressing syphilis screening, found that 27% of their convenience sample spontaneously mentioned the campaign with no prompting and an additional 44% remembered the campaign when prompted. With another campaign addressing syphilis screening, Stephens et al. [25] indicated that 33% of their convenience sample reported some recall of the campaign, with around half of these recalling the campaign spontaneously, and 44% recalled the campaign when prompted with a visual aid.

Against this background of promising but partial evidence (and with a recognition of the common design challenges which accompany evaluating mass media campaigns), we report an outcome evaluation of the Make Your Position Clear (MYPC) mass media campaign. In this paper, we focus on our two principal research questions: 1. What was the extent of self-reported exposure to the MYPC campaign among men frequenting venues for gay men and MSM? 2. Did sexual health related behaviours (i.e., unprotected anal intercourse (UAI), HIV testing and STI testing and use of appropriate lubricant) vary by degree of exposure to the campaign?

## Methods

### The campaign

Make Your Position Clear (MYPC) was funded by an alliance of health boards across the West of Scotland. It ran from October 2009 to July 2010. It was described by the project group formed to represent the health boards as a sexual health social marketing campaign aimed at MSM (including those who identified as gay or bisexual) [26]. It had two key aims: to promote the use of condoms and water-based lubricant with each episode of anal intercourse; and to promote regular sexual health check ups and HIV testing every 6 months, or more often if the individual had put himself at risk. The development of the campaign involved consultation with voluntary sector agencies and representatives of the target group (through focus groups), prior to commissioning a creative agency to develop the materials. The consultations and focus groups centred on obtaining views on setting, medium, imagery and tone. The first set of posters and images produced were subjected to further consultation with the voluntary sector agencies, and revisions were prepared in line with feedback.

Six related images were used in the campaign materials: four were designed for display in venues and websites used by or targeted at MSM, and two were designed for display in other venues. All images included two men and a ‘position’ name and number (e.g., “Position #21, the watercooler”), with one of the key messages (“Whatever position you’re in, it’s a lot safer with condoms and lube” or “Whatever position you’re in, sexual health check ups have a part to play”) and a link to the campaign website [26, appendix 7]. As far as we are aware, there was no explicit attempt to use any theoretical behaviour change techniques within the campaign development, and we were completely independent of the development and implementation of the intervention.

Campaign materials included posters, electronic images and leaflets, with a dedicated campaign website. Posters and leaflets were distributed to GP practices, dental surgeries, community pharmacies, sexual health clinics, community centres and libraries across all three health boards. Within the health board covering Glasgow, the posters and leaflets were also distributed to bars, clubs and saunas targeted at MSM and gay men (i.e., the “gay scene”), further education establishments and sports centres. Outreach workers from a local voluntary sector agency were involved in the distribution of leaflets at bars, clubs and saunas targeted at gay men and other MSM. Posters were displayed on local buses and on the Glasgow subway trains, and at some local authority workplaces (including certain fire and police stations). Materials were also shown and distributed at the Pride 2010 event in Glasgow. Online, the campaign was advertised on five sites, two of which were sites targeted at MSM. A smart phone application designed for MSM also advertised the campaign. Overall, the website received 9557 hits, 2813 of which were from people in Scotland [26].

### Design and procedure

The current study involved one cross-sectional survey of men recruited from seven bars frequented by gay men and other MSM in Glasgow in July 2010, ten months after the campaign had been launched (i.e., post-test only). We surveyed bars at two different time points: in the early evening (19.00-21.00) and the late evening (21.00-23.00). No bar was visited twice in the same evening. At the end of the survey period each bar had been visited at both time points on each day of the week. A team of temporary fieldworkers was trained then employed to distribute and collect anonymous, self-complete questionnaires in the bars. All men present or entering the venue were approached to complete a questionnaire. Fieldworkers completed forms indicating the number of men who agreed and declined to participate, and the number of men who had already participated and declined. This enabled the accurate calculation of response rates. Ethical approval was granted by the Psychology Ethics Subcommittee at Glasgow Caledonian University.

### The measures

The questionnaire included items to assess demographic and contextual variables (see Table 1), sexual behaviour (numbers of sexual contacts, anal intercourse [AI] sexual partners, and unprotected anal intercourse [UAI] partners in the previous 12 months), sexual health behaviours (recency of HIV testing, recency of STI testing, the correct use of lubricant in anal sex, and self reported STI diagnosis in the previous 12 months). In addition two approaches to measuring risk of HIV transmission were developed, one simple and one complex. The simple measure categorised the number of UAI partners (0; 1; or ≥2) reported within the 12 months preceding data collection. The complex measure incorporated risk reduction strategies that included avoiding anal sex, perceived HIV status of UAI partners and perceived serosorting (selecting UAI partners on the basis of shared HIV negative status). The complex measure of risk categorised men as having either 1) no AI partners at all; 2) ≥1 AI partners but no UAI partners; 3) ≥1 UAI partners but no partners were casual, all were of known HIV status, and no partners were HIV positive; and 4) ≥1 UAI partners and partners were casual, and/or their HIV status was unknown, and/or partners might have been HIV positive.

**Table 1:** Description of sample: maximum n = 784, excluding those self-identified as HIV positive

		**n**	**%**
Age in years (n = 758)	≤24	213	28.1
	25-34	238	31.4
	35-44	204	26.9
	≥45	103	13.6
Education (n = 759)	Secondary	137	18.1
	Vocational/Further education	318	41.9
	Degree/Postgraduate	304	40.1
Employment (n = 768)	Employed/Self-employed	613	79.8
	Unemployed/Student/retired	155	20.2
Area of residence (n = 737)	Glasgow	551	74.8
	Edinburgh	11	1.5
	Rest of Scotland	134	18.2
	Rest of UK	38	5.2
	Elsewhere	3	0.4
Frequency of going out on the “gay scene” (n = 764)	≤ once per month	185	24.2
	2-3 times per month	226	29.6
	1-2 times per week	256	33.5
	≥ 3 times per week	97	12.7
Number of UAI partners in previous year (n = 762)	0	402	52.8
	1	247	32.4
	≥2	113	14.8
AI/UAI risk in previous year (n = 736) ^a^			
	No AI or UAI partners in previous year	138	18.8
	≥1 AI partners, no UAI partners	248	33.7
	≥1 UAI partners, no additional risk factors	112	15.2
	≥1 UAI partners, ≥1 additional risk factors	238	32.3
Use of lubricant with anal intercourse in previous year (n = 621)	Water- or silicone-based lubricant only	457	73.6
	Any other practice	164	26.4
Recency of HIV testing (n = 766)	< 6 months previously	304	39.7
	6-12 months previously	134	17.5
	1-5 years previously	127	16.6
	> 5 years previously	47	6.1
	Never	154	20.1
Recency of STI testing (n = 740)	< 6 months previously	272	36.8
	6-12 months previously	124	16.8
	> 12 months previously	206	27.8
	Never	138	18.6
Intention to have HIV test in following 6 months (n = 731)	Strongly agree/Agree	472	64.6
	Uncertain/Disagree/Strongly Disagree	259	35.4

Notes. *UAI* unprotected anal intercourse, *AI* anal intercourse.

^a^ Additional risk factors: whether any UAI partners had been casual, whether the HIV status of these partners was known, and whether any had been HIV positive.

Exposure and reach of the campaign were measured as follows (see also Table 2): unaided recall (“In the last 12 months have you seen any adverts, posters or leaflets that provided information about sexual health issues or campaigns?”: if “yes”, the respondent was asked to write down the names of up to three campaigns); recognition of the MYPC campaign, with presentation of example images (“Have you heard of the ‘make your position clear’ campaign?”; “Do you recognise the ‘make your position clear’ logo?”; “Have you seen these or other ‘positions’ images like them in the last 12 months?”); frequency of having seen MYPC images (“How often have you seen the images ON the gay scene?” and “How often have you seen the images OUTSIDE the scene?”); number of places or sites recalled in which the MYPC images had been seen (with a list of 14 sites provided, including the MYPC website: see Table 3); engagement (“Did you pick up any of the ‘make your position clear’ campaign leaflets?”; “Did you talk to an outreach worker about the ‘make your position clear’ campaign?”); recognition of the key messages of the campaign (“What do you think the ‘make your position clear’ campaign is about?”).

**Table 2:** Exposure to the ‘make your position clear’ (MYPC) campaign: maximum n = 784, excluding those self-identified as HIV positive

		**n**	**%**
Unaided recall of campaign (n = 784)	Named/alluded to MYPC	56	7.1
	Named other campaigns only	203	25.9
	No recall of campaigns/omitted	525	67.0
Heard of MYPC campaign? (n = 751)	Yes	261	34.8
	No	438	58.3
	Unsure	52	6.9
Recognise MYPC logo? (n = 754)	Yes	257	34.1
	No	462	61.3
	Unsure	35	4.6
Seen MYPC images in previous 12 months? (n = 742)	Yes	424	57.1
	No	318	42.9
If images seen: How often seen images on gay scene? (n = 378)	Never	76	20.1
	Occasionally	188	49.7
	Many times	114	30.2
If images seen: How often seen images outside the gay scene? (n = 371)	Never	137	36.9
	Occasionally	147	39.6
	Many times	87	23.5
Picked up MYPC leaflet? (n = 671)	Yes	68	10.1
	No	603	89.9
Talked to outreach worker about MYPC? (n = 729)	Yes	27	3.7
	No/unsure	702	96.3

**Table 3:** Places where ‘make your position clear’ (MYPC) campaign materials had been seen: maximum n = 424, excluding those self-identified as HIV positive and those who had not seen any MYPC images in the previous year

**Places where MYPC materials seen**	**‘Yes’ responses**	
	**n**	**%**
In a bar or club	259	68.9
Greens or LA Fitness [private health clubs]	31	8.5
In a public gym/sports centre	33	9.0
On the Glasgow subway	189	50.9
On a bus	107	28.8
At work, college or university	61	16.4
In a pharmacy	45	12.1
In a public library or community centre	24	6.5
‘Make your position clear’ campaign website	45	12.2
Online banner (e.g. on Facebook or Gaydar)	122	33.2
At Pride	125	34.0
Sexual health or GUM clinic ^a^	102	27.6
In a GP surgery or practice ^a^	74	19.8
In a sauna ^a^	53	14.4

Notes. ^a^ indicates excluded from measure of exposure to the campaign because a site of HIV testing.

An overall measure of degree of exposure to the MYPC campaign was developed using responses to the following questions: unaided recall of the MYPC campaign; the three MYPC recognition questions; frequency of seeing the images on and off the “gay scene”; number of places/sites recalled in which the MYPC materials had been seen; the two engagement questions; and recognition of the key campaign messages. Note that potential sites of HIV testing (saunas, GP surgeries and practices, and sexual health/GUM clinics) were excluded from the measure of places/sites in which MYPC materials had been seen (see Table 3) and thus did not contribute to the measure of exposure.

Those who did not recall the MYPC campaign unaided and gave negative responses to the three recognition questions were classed as having had no exposure to the campaign. Remaining scores varied between 1 and 25, and respondents were grouped as follows: low exposure (score 1–6); mid exposure (7–10); and high exposure (≥11). Twenty-two respondents did not provide sufficient data to permit classification.

### Statistical analysis

The analysis was conducted using SPSS 18.0 for Mac. Differences between campaign exposure groups on the measures of interest were initially explored using Mantel-Haenszel linear-by-linear chi-square analysis (χ^2^). Following this, binary logistic regression was used to examine whether there were differences between categories of behaviour according to campaign exposure when adjusted for other relevant variables. As some of our dependent variables focused upon HIV testing (such as intentions to have an HIV test and our complex measure of HIV transmission risk), those who identified themselves as HIV positive (through their response to a question on the result of the most recent HIV test) were excluded from the analysis reported here.

## Results

### Characteristics of the sample

In total, 1313 men were approached and 822 participated (a response rate of 62.6%). The maximum sample included in the analysis was 784, excluding 38 men who identified themselves as HIV positive. An overview of the sample characteristics is presented in Table 1. The mean age of the sample was 32 years (SD = 10.52), ranging from 18 to 68 years. Most were employed or self-employed (80%, n = 613), and a substantial number were educated to university level (40%, n = 304). Just under 75% of the sample (n = 551) resided in Glasgow or the surrounding areas. With regard to sexual behaviour, 53% (n = 402) reported no UAI partners within the previous year, while 15% (n = 113) reported two or more: however, 32% (n = 238) of those providing the relevant information reported at least one UAI partner in the previous year with at least one additional risk factor (a casual partner, a partner of unknown HIV status, or a partner known to be HIV positive).

### Exposure to the MYPC campaign

Tables 2 and 3 shows exposure to the MYPC campaign. A total of 34.8% of the sample had heard of MYPC, and 34.1% recognised the logo. Of those who indicated that they had seen the MYPC images in the previous 12 months (57.1%), 79.9% reported seeing the images on the gay scene, and 63.1% had seen the images in other places. Recall of engagement with the campaign materials was low: 10.1% had picked up MYPC leaflets and 3.7% had talked to an outreach worker about the MYPC campaign. Of those who indicated that they recognised the MYPC images, 12.2% (n = 45) indicated that they had visited the MYPC campaign website.

As described above, four categories of respondent were identified: those with no exposure to the campaign (39.9%, n = 304); those with a low level of exposure (19.2%, n = 146); those with a mid level of exposure (23.0%, n = 175); and those with high exposure (18.0%, n = 137). No significant differences were found between the four groups in terms of age, employment status or educational qualifications. As would be expected, those who did not reside in the Glasgow area had less exposure to the campaign; 55.5% (n = 101) of those who resided elsewhere had had no exposure, compared with 33.6% (n = 180) of those residing in the Glasgow area: χ^2^ (1, n = 717) = 26.81, p < .001. Use of the gay scene was also related to exposure; those who used the gay scene more than once per week had greater exposure to the campaign than those who used the scene less frequently: χ^2^ (1, n = 742) =29.14, p < .001.

### MYPC campaign exposure and target sexual health behaviours

The cross-tabulations between campaign exposure and the target behaviours (sexual and health-related) are shown in Table 4. Neither the simple nor complex measure of HIV risk behaviour varied significantly in line with the categories of campaign exposure (both p > .1). However, significant variation was noted on the remaining four target behaviours. Those having been tested for HIV within the previous six months were more likely than other respondents to have had mid or high exposure to the campaign (p < .001), and similar patterns were noted with regard to STI testing (p = .009) and intention to be tested for HIV (p = .028). Finally, those reporting having used inappropriate forms of lubricants were more likely to have had no exposure to the MYPC campaign than those who reported having used only appropriate lubricant (p = .015).

**Table 4:** **Sexual and health related behaviours by categories of exposure to the MYPC campaign (Mantel-Haenszel Linear Association, χ**^**2**^**)**

	**No MYPC exposure (N = 304, 39.9%)**	**Low MYPC exposure (N = 146, 19.2%)**	**Mid MYPC exposure (N = 175, 23.0%)**	**High MYPC exposure (N = 137, 18.0%)**		
	**N (row%)**	**N (row%)**	**N (row%)**	**N (row%)**	**χ**^**2**^	**p**
Number of UAI partners in previous year					0.63	.428
0	157 (40.2)	82 (21.0)	87 (22.3)	65 (16.6)		
1	98 (40.7)	40 (16.6)	56 (23.2)	47 (19.5)		
≥2	41 (36.9)	22 (19.8)	29 (26.1)	19 (17.9)		
AI/UAI risk ^a^					0.28	.596
No AI or UAI partners in previous year	53 (39.6)	35 (26.1)	24 (17.9)	22 (16.4)		
≥1 AI partners, no UAI partners	96 (39.8)	44 (18.3)	60 (24.9)	41 (17.0)		
≥1 UAI partners, no additional risk factors	36 (33.3)	18 (16.7)	28 (25.9))	26 (24.1)		
≥1 UAI partners, ≥1 additional risk factors	97 (41.5)	41 (17.5)	56 (23.9)	40 (17.1)		
Use of lubricant with anal intercourse in previous year					5.97	.015
Water- or silicone-based lubricant only	157 (35.4)	88 (19.8)	115 (25.9)	84 (18.9)		
Any other practice	82 (50.9)	20 (12.4)	32 (19.9)	27 (16.8)		
HIV testing					17.36	<.001
< 6 months previously	99 (33.6)	44 (14.9)	83 (28.1)	69 (23.4)		
> 6 months previously or never	199 (43.5)	100 (21.9)	91 (19.9)	67 (14.7)		
STI testing					6.83	.009
< 6 months previously	97 (36.2)	40 (14.9)	68 (25.4)	63 (23.5)		
> 6 months previously or never	188 (40.5)	100 (21.6)	103 (22.2)	73 (15.7)		
Intention to have HIV test in following 6 months					4.84	.028
Strongly agree/Agree	179 (38.4)	75 (16.1)	119 (25.5)	93 (20.0)		
Uncertain/Disagree/Strongly Disagree	108 (42.0)	61 (23.7)	49 (19.1)	39 (15.2)		

Notes. Numbers vary in line with missing data.

^a^ Risk factors: whether any UAI partners had been casual, whether the HIV status of these partners was known, and whether any had been HIV positive.

Further binary logistic regression analyses were conducted in order to control for other factors that could account for the differences in the target behaviours relating to testing and lubricant use. Area of residence (Glasgow area; elsewhere) and use of the gay scene (≤ once per month; 2–3 times per month; ≥ once per week) were included in these analyses because both were related to the degree of campaign exposure. Age was also included: although it was not related to campaign exposure, it was significantly related to HIV and STI testing, and also to intention to have an HIV test, such that younger men were more likely to have tested and to have a stronger intention to test. Each target sexual health behaviour is discussed in turn below (n = 784 for each analysis).

*Recency of HIV testing* When adjusted for age, area of residence, and use of the gay scene, MYPC campaign exposure still significantly differentiated between those who had been tested for HIV within the previous six months and those who had not; those with no or low exposure were significantly less likely to have been tested than those with mid or high exposure (see Table 5): for the comparison between high exposure and no exposure, adjusted odds ratio (AOR) = 1.96, 95% CI = 1.26 to 3.06, p = .003. Age also significantly discriminated between the groups, such that those who had recently tested were significantly younger than those who had not: AOR = 0.97, 95% CI = 0.95 to 0.98, p < .001.

**Table 5:** Sexual health variables by categories of MYPC exposure, adjusted for age, area of residence and use of the gay scene through binary logistic regression: adjusted odds ratios (AOR), 95% Confidence Intervals (CI) and p-values

**Tested for HIV within previous 6 months**				
		**AOR**	**95% CI**	**p**
Age		0.97	0.95-0.98	<.001
Area of residence	Glasgow	1.06	0.73-1.54	.759
Gay scene use	≤ 1 per month	0.92	0.61-1.38	.683
	2-3 per month	0.94	0.65-1.36	.741
MYPC exposure	Low exposure	0.90	0.56-1.42	.638
	Mid exposure	1.89	1.24-2.87	.003
	High exposure	1.96	1.26-3.06	.003
Tested for STIs within previous 6 months				
		AOR	95% CI	p
Age		0.97	0.95-0.98	<.001
Area of residence	Glasgow	0.97	0.66-1.42	.866
Gay scene use	≤ 1 per month	0.78	0.51-1.18	.231
	2-3 per month	0.70	0.48-1.02	.061
MYPC exposure	Low exposure	0.73	0.45-1.17	.186
	Mid exposure	1.20	0.78-1.84	.409
	High exposure	1.37	0.88-2.16	.167
Intention to test for HIV within following 6 months				
		AOR	95% CI	p
Age		0.94	0.93-0.96	<.001
Area of residence	Glasgow	1.54	1.05-2.26	.028
Gay scene use	≤ 1 per month	0.68	0.45-1.05	.080
	2-3 per month	0.96	0.64-1.43	.837
MYPC exposure	No exposure	1.61	1.02-2.55	.042
	Mid exposure	2.01	1.20-3.36	.008
	High exposure	1.68	0.98-2.90	.060
Intention to test for HIV within following 6 months (adjusted for recency of HIV testing)				
		AOR	95% CI	p
Tested for HIV within previous 6 months		4.38	2.92-6.55	<.001
Age		0.95	0.93-0.96	<.001
Area of residence	Glasgow	1.54	1.03-2.31	.038
Gay scene use	≤ 1 per month	0.66	0.42-1.03	.067
	2-3 per month	0.98	0.64-1.49	.916
MYPC exposure	No exposure	1.54	0.95-2.49	.067
	Mid exposure	1.55	0.90-2.66	.116
	High exposure	1.30	0.73-2.32	.367
Use of appropriate lubricant with anal sex in previous year				
		AOR	95% CI	p
Age		1.01	0.99-1.03	.201
Area of residence	Glasgow	1.40	0.91-2.16	.124
Gay scene use	≤ 1 per month	1.00	0.62-1.63	.993
	2-3 per month	1.27	0.81-2.00	.303
MYPC exposure	No exposure	0.42	0.23-0.77	.005
	Mid exposure	0.71	0.37-1.37	.303
	High exposure	0.64	0.32-1.28	.205

Notes. Reference category for area of residence is other than Glasgow; reference category for gay scene use is ≥ once per week; reference category for MYPC exposure is either no exposure (HIV and STI testing) or low exposure (HIV testing intention and lubricant use).

*Recency of STI testing* As can be seen from Table 5, when adjusted for age, area of residence and use of the gay scene, those who had recently been tested for STIs no longer differed on levels of campaign exposure: for the comparison between high exposure and no exposure, AOR = 1.37, 95% CI = 0.88 to 2.16, p = .167.

*Intentions to take an HIV test* When adjusted for age, area of residence and use of the gay scene, those with mid campaign exposure were somewhat more likely than those with low campaign exposure to have strong intentions to seek HIV testing in the following six months. Age and area of residence independently distinguished between the groups, such that those with strong intention to be tested for HIV were younger, and were also more likely to reside in the Glasgow area. A second analysis was conducted adjusting for previous HIV testing; at this stage, campaign exposure no longer significantly differentiated between the intention groups: for the comparison between high exposure and no exposure, AOR = 1.30, 95% CI = 0.73 to 2.32, p = .367.

*The correct use of lubricant in anal sex* When adjusted for age, area of residence and use of the gay scene (see Table 5), those with no campaign exposure were significantly less likely to always use appropriate lubricant than those with low exposure: AOR = 0.42, 95% CI = 0.23 to 0.77, p = .005. However, those with high exposure did not differ significantly from those with low exposure.

## Discussion

The primary research question we addressed concerned exposure to the campaign among men frequenting venues for gay men and other MSM in Glasgow. Our secondary research question explored the impact of the campaign. In regard to the primary research question, 60.1% of the sample reported some exposure to the campaign: the degree of this exposure ranged from the 7.1% who recalled the campaign unaided, through the 34% to 35% who, when prompted, had heard of the campaign or recognised the logo, to the 57.1% who recognised the example of the poster shown to them. In the wider literature concerning mass media HIV prevention campaigns (targeting various populations with various media sources) exposure usually ranges between 52% and 77% [11]. Thus, by this benchmark, MYPC had fairly standard reach. The indicators of exposure were lower than those noted by Martínez-Donate et al. [23] and Plant et al. [24], but it is worth noting that the campaign evaluated by Plant et al. had been running for a number of years and the developers of it had focussed particularly upon brand recognition. Critically the overall measure of exposure operationalised here captured some sense of the frequency of exposure in addition to a range of aspects relating to both campaign recollection (unaided and with prompts) and behaviours indicative of active engagement with MYPC (such as talking to outreach workers about the campaign). As such the measure of exposure was an improvement on the typical approach used within most evaluative research (which usually uses dichotomous measures of exposure [27]).

Within the constraints of the post-test research design, this approach to measuring exposure raised key issues and questions. It was not possible to treat exposure as a continuous variable (and therefore to examine dose–response relationships) because around 40% of the sample were classed as having had no exposure at all. However, even within this constraint, there was little indication that greater exposure was associated with improved sexual health practice: those who had tested for HIV within the previous six months were likely to have had mid or high exposure to the campaign (as opposed to none), but those with high exposure were not more likely to have been tested for HIV than those with mid exposure. We excluded sites of HIV testing (including saunas) from the overall measure of exposure, but it remains possible that men who were tested for HIV during the campaign were alerted to the campaign when tested, such that images became more salient (leading to greater self-reported campaign exposure). These men might also have been more concerned with sexual health within the gay and MSM communities to begin with, and therefore more likely to engage with any relevant campaign materials. This is consistent with the finding that intention to be tested for HIV did not vary according to campaign exposure when previous HIV testing had been taken into account. However, if this were the sole explanation of the results, we would expect to find that STI testing also varied in line with campaign exposure, and this was not the case when adjusted for age, area of residence and use of the gay scene. This discrepancy is, in fact, consistent with the findings of other authors [19]. In summary the findings presented here resonate with the literature, in that there were significant differences between the categories of exposure in relation to recency of HIV (but not STI) testing and the appropriate use of lubricant in anal sex. Whilst only a better research design (e.g., a randomised control trial) could illuminate the meaning of these differences, in combination with the emerging evidence base relating to both the effectiveness of mass media more broadly [13] and the importance of combination HIV prevention in general [5], they make a contribution to the existing evidence base.

There are, of course, a number of limitations to the study. Overall, the findings suggest some potential impact of the campaign. Yet the cross-sectional nature of the data means that the findings represent an explanatory *cul de sac*; further, given the limited range of variables included within this analysis, there is no way of knowing the importance of other (unmeasured) variables in explaining variance within the sexual health behaviours, or given the cross-sectional nature of the data set, the interpretation and meaning of mediating effects [28]. The recruitment strategy we used meant that we sampled men frequenting the most prominent gay venues in Glasgow: while most of these men probably identified as gay (rather than as MSM), it was not possible to examine the role of identification in the analysis, and it was not possible using this strategy to include men who did not use or participate in the gay scene in Glasgow.

The current economic climate could result in a lack of funding to support gold standard evaluations (i.e., RCTs) of mass media interventions promoting health behaviour change, making it highly likely that other researchers will be evaluating interventions within similar constraints. In relation to the use of theory within mass media campaigns, there is much room for improvement. Noar et al. [11] highlight the need to think carefully about the use of theory within mass media techniques. Critically they draw an important distinction between the ways theories can help inform content and delivery. In terms of the former, there is a burgeoning literature concerning behavioural change techniques, their theoretical basis [29], and ways of retrospectively assessing their implementation and role within evidence-based practice [23]. Yet in relation to theories of message delivery which can focus upon persuasion, information processing, and emotional appeals [30-32], there is far less work and critical engagement. There is a need for research which explicitly and rigorously focuses upon the best evidence regarding which specific delivery techniques are most effective, in which contexts, and, more broadly, how to systematically code for modes of intervention delivery and how to grapple with the particular challenges of combination prevention and its particular dialogical synergistic effects. Further, we need to understand, account for and avoid any possible negative or undesirable effects of campaigns and interventions: men who are already protecting the sexual health of themselves and their partners are likely to have greater exposure to relevant social marketing campaigns than other men (either through displays of material at sexual health clinics, or through actively seeking out such campaigns), and the point at which campaigns begin to degrade motivation is not yet well documented.

## Conclusions

In summary, this paper has highlighted a series of associations between exposure to a sexual health promotion mass media campaign and sexual health behaviours. Whilst the results are intriguing they are also frustrating. As available funding for the comprehensive evaluation of complex interventions has decreased, the demand for demonstrable intervention evaluation (usually from those funding the interventions) has increased. Within this challenging context, increasing numbers of researchers will be working on ‘pragmatic’ evaluations, such as the study presented here, which employ inherently weak (i.e., non-experimental) evaluative designs. To compensate, there is scope for reliance upon a better interrogation of the existing high quality evidence in terms of the role of theory, or as we have suggested, the detail and complexity of examining the role of intervention delivery. Equally, there is scope to examine key processes involved in mass media consumption and sexual health behaviour change in more detail. Ideally, this could be addressed through programmatic mixed methods research (such as combinations of controlled experimental studies with inductively based qualitative research examining aspects of process evaluation). These could focus upon, for example, theorising campaign exposure in relation to individual change: intra-subjective variation in perceptions of risk across time, the role of location and its relationship to sexual and health salience. Alternatively, examining individual differences presents another possibly fruitful approach; for example, exploring the role of concepts such as sexual health literacy, social capital or capability in shaping men’s *a priori* sexual health engagement and the salience of mass media campaigns.

## Competing interests

The authors declare that they have no competing interests.

## Authors’ contributions

PF took responsibility for drafting the manuscript; CK conducted the analysis, contributed to the drafting of the manuscript and took responsibility for the revision; LM contributed to the drafting of the manuscript. All authors contributed to the design, planning and coordination of the study, and all authors read and approved the final manuscript.

## Pre-publication history

The pre-publication history for this paper can be accessed here:

http://www.biomedcentral.com/1471-2458/13/737/prepub
